# Exosomes derived from LPS-stimulated human thymic mesenchymal stromal cells enhance inflammation via thrombospondin-1

**DOI:** 10.1042/BSR20203573

**Published:** 2021-10-12

**Authors:** Qianru Li, Jing Li, Lei Sun, Yun Sun, Fei Zhao, Pingping Liu, Xin Peng, Xiaoyan Xuan, Yun Li, Peng Wang, Chen Tan, Ying Du

**Affiliations:** 1 Department of Immunology, School of Basic Medical, Zhengzhou University, Zhengzhou, China; 2 Clinical Laboratory, The First Affiliated Hospital of Henan University of CM, Zhengzhou, China

**Keywords:** exosome, inflammation, thrombospondin-1, Thymic mesenchymal stem cells

## Abstract

Inflammatory response mediated by immune cells is either directly or indirectly regulated by mesenchymal stromal cells (MSCs). Accumulating evidence suggests that thrombospondin-1 (TSP-1) is highly expressed in response to inflammation. In this work, we isolated and identified human thymic mesenchymal stromal cells (tMSCs) and detected the expression of TSP-1. We found that tMSCs expressed TSP-1 and Poly (I:C) or LPS treatment promoted the expression of TSP-1. Further, we isolated and identified exosomes originating from tMSCs (MEXs). Notably, exosomes derived from LPS-pretreated tMSCs (MEXs^LPS^) promoted the polarization of macrophages to M1-like phenotype and IL-6, TNF-α secretion as well as the pro-inflammatory differentiation of CD4^+^T cells into Th17 cells. Upon silencing the expression of TSP-1 in tMSCs, the pro-inflammatory effects of MEXs^LPS^ were suppressed. Therefore, these findings uncovered TSP-1 as the principal factor in MEXs^LPS^ pro-inflammatory regulation.

## Introduction

Mesenchymal stromal cells (MSCs) are adult, fibroblast-like multipotent cells that widely exist in a variety of body tissues. MSCs participate in immune regulation and tissue repair. Although a challenge has faced that how to define such a population of cells, it is clear that some populations of MSCs are capable of exhibiting stem cell function *in vivo* [1]. By regulating the proliferation, differentiation and functional status of immune cells, tMSCs consequently regulate the secretion of inflammatory cytokines [1,2], Thus, tMSCs have been shown to exhibit great potential in regenerative medicine and immunotherapy of inflammation-related diseases [3]. Notably, the immune regulation of MSCs has strong plasticity and its outcome is influenced by several factors, such as MSCs concentration (the ratio of MSC to immune cells), direct or indirect cell contact, and whether MSCs have been pretreated [4]. Therapeutic efficacy of MSCs thus is improved in type IV hypersensitivity, liver injury, colitis, graft-versus-host disease, and arthritis via interferon (IFN)-γ or tumor necrosis factor (TNF)-α combined with interleukin (IL)-1 [5,6]. However, at stages of illness, the low inflammation levels significantly reduce the therapeutic effect of MSCs and can promote the development of the disease [7]. When no inflammation arises or is in a low state, MSCs show reduced effectivity in GvHD treatment [8,9]. Similarly, the therapeutic effect is weakened in experimental autoimmune encephalomyelitis when MSCs are transplanted during disease remission [10,11]. Based on these observations, MSCs can either promote or suppress immune responses. It is worth noting that the inflammatory state determines the fate of the immune regulation of MSCs.

Thrombospondin 1 (TSP-1) is the prime member of thrombospondin (TSP) family. A variety of normal cells, including macrophages, endothelial cells, among others, secrete TSP-1 [12]. TSP-1 has been found to maintain vascular structure and homeostasis by regulating biological functions such as cell proliferation [13], apoptosis and adhesion [14]. Additionally, TSP-1 function as a chemo-attractant, impacting on various inflammatory cells [14]. High levels of TSP-1 expression have been implicated in conditions associated with tissue damage and inflammation [15,16]. TSP-1 is considered as an inflammation marker and participates in early fibrosis in human glomerulopathies [17]. However, the TSP-1 gene deletion generates mixed effects on the inflammatory responses depending on different disease models. For instance, the development of diabetic nephropathy was found to be reduced in TSP-1-deficient mice as demonstrated by reduced renal infiltration with inflammatory cells [18]. Moreover, using a diet-induced obesity mouse model, a lack of TSP-1 was revealed to potentially reduce obesity-related inflammation and improve insulin sensitivity [19]. This is via a reduction in adhesion, migration and inflammation signals in TSP-1^−/−^macrophages [19]. In a different study, TSP-1 deficient mice exhibited acute pneumonia, leukocytosis, pancreatitis and lacrimal inflammatory infiltration [20]. Ng et al. in their work reported that, in the retina, TSP-1 mediated pro-inflammatory microglia such that a lack of TSP-1 was associated with a spontaneous increase in inflammatory mediators [21]. Further, subcutaneous air pouch and systemic candidiasis models demonstrated that endogenous TSP-1 promotes activation of inflammatory macrophages [22]. TSP-1 triggers macrophage IL-1 production [23] and IL-10 production, promotes resolution of experimental lung injury [24] and plays a significant role in regulation of migration and adhesion of mononuclear cells in aortic aneurysm [25].

Exosomes are small (40 and 120 nm) membranous vesicles of endocytic origin produced by all types of cells [26]; they exist in a variety of body fluids [27,28]. Exosomes can be immediately ingested by local tissues or gathered in body fluids, eventually affect distant target organs [29]. Increasing evidence has recently suggested that exosomes participate in the regulation of inflammation, fibrosis, and proliferation as well as the migration of vascular endothelial cells, all of which are crucial in the repair of damaged tissues [30]. Exosomes contain numerous signaling molecules, including nucleic acids, proteins and lipids, and play an important role in intercellular communication. [31,32]. Recent assessments have shown that exosomes released by MSCs can prevent tissue damage [33–35]. *In vitro*, MSC-derived exosomes show an inflammatory inhibitory regulatory effect consistent with that of MSCs [36]. Indeed, MSC-derived exosomes can recapitulate MSC’s efficacy, whereas transplanted MSCs can mediate their efficacy *in vivo* through secreted exosomes [37]. It has also been shown that exosomes derived from MSCs can ameliorate Severe, steroid-resistant asthma (SSRA) by moderating inflammation, it is achieved by reshaping macrophage polarization via inhibition of Traf1 [38]. Moreover, TSP-1 derived from OSCC exosomes promote the polarization of macrophages to M1-like phenotype, an indication that exosomes are an important channels for TSP-1 transport [39].

Earlier findings from our work showed significantly higher TSP-1 expression in myasthenia gravis (MG) thymus. In MG thymus, an infection-like inflammatory response can occur, this can result in germinal centers [40]. In the present study, we isolated human thymic mesenchymal stromal cells (tMSC) and found that lipopolysaccharides (LPS) or polyinosinic-polycytidylic acid (Poly (I:C)) potentially stimulated tMSC to up-regulate TSP-1 expression. However, whether TSP-1 potentially impacted on the immunomodulatory function of tMSCs and its exosomes is poorly understood. This prompted us to further explore the role of TSP-1 in tMSCs and tMSCs exosomes in regulating the inflammatory response of immune cells via the alteration of TSP-1 expression in tMSCs. Herein, we aimed to investigate the phenotypic alterations of macrophages in the inflammatory conditions through tMSCs exosomes-transferred TSP-1.

## Material and methods

### tMSCs culture

We obtained tMSCs from the normal thymus of children with congenital heart disease admitted at the Second Affiliated Hospital of Zhengzhou University. Informed consent from the parents of the children was obtained before commencing the experiments. The study was approved by the Life Science Ethics Review Committee of Zhengzhou University. tMSCs were cultured in DMEM (Solarbio) supplemented with 10% FBS, penicillin (100 U/ml) and streptomycin (100 U/ml) in 37°C/5% CO_2_. Cells were characterized based on the expression of common MSC markers CD105, CD90, CD73 (Biolegend) by flow cytometry. tMSCs (P3-P6) cells were used in the experiment.

### TSP-1-knockdown lentivirus preparation

Stable TSP-1 knockdown cells for tMSCs were generated via transfection with TSP-1-specific short hairpin RNA (SHCLND-NM_003246.2-3029s21c1 for sh1, SHCLND-NM_003246.2-3071s21c1 for sh2, Sigma-Aldrich) lentivirus, and positively selected with ampicillin (10 µg/ml, Solarbio).

### Human cells stimulation

tMSCs (up to 70% confluence) were pretreated with LPS (1 μg/ml, Solarbio) or Poly (I:C) (50 μg/ml, Invivogen) in 24, 48 and 72 h, and the expression of TSP-1 was detect in tMSCs by quantitative real-time PCR and Western blot. tMSCs were infected with TSP-1 knockdown lentivirus for 72 h, and the expression of TSP-1 was detected in tMSCs.

### Conditioned media preparation

To generate conditioned media for the cells, tMSCs (up to 70% confluence) were cultured for 48 h in DMEM media with exosome-free FBS (Umibio). The cell-free supernatants were collected and centrifuged at 500 ***g*** for 10 min at 4°C, and at 3000 ***g*** for 10 min at 4°C. The conditioned media was finally obtained after filtration through a 0.22 μm filter.

### Exosome isolation

The collected conditioned media was centrifuged at 2000 ***g*** for 20 min at 4°C to remove dead cells and cell debris then transferred to Amicon® Ultra-15 centrifugal filter device (100 kD, Millipore) and concentrated by centrifugation at 5000 ***g*** for 30 min at 4°C. After filtration through a 0.22 μm filter, exosomes were isolated using Exosome Isolation Kit (Umibio) and resuspended in PBS. The extracted coarse exosomes were transferred to Exosome Purification Filter Column (Umibio) and centrifuged at 3000 ***g*** for 10 min at 4°C to obtain the purified Exosome particles for validation or subsequent experiments.

### Exosome identification

Exosomes were characterized based on the expression of exosomes markers by flow cytometry. Extracted exosomes (10 μg) were incubated with 1 µl Aldehyde/Sulfate Latex Beads (Invitrogen) 15 min at RT. After washed, exosomes-beads complex were incubated with Anti-CD63-PE, Anti-CD81-APC, Anti-CD9-PE (Biolegend). The morphology of exosomes was observed using the transmission electron microscopy (TEM). About 20 μl exosome suspensions were dropped on a sample-loaded copper mesh. Samples were negatively stained with 2% uranyl acetate solution and air dried for 30 min.

### Macrophage differentiation and polarization

THP-1 cells were obtained from ATCC and cultured in RPMI 1640 media supplemented with 10% FBS, penicillin (100 U/ml) and streptomycin (100 U/ml). To obtain resting macrophages (M0), THP-1 cells were differentiated under phorbol-12-myristate-13-acetate (PMA, 100 ng/ml, Sigma-Aldrich) for 24 h. IFN-γ (20 ng/ml, PeproTech) and LPS (20 ng/ml, Solarbio) were used to activate M0 cells. Morphology of THP-1 derived macrophages was observed under an optical microscope and micrographs were taken.

Normal human peripheral blood mononuclear cells (PBMC) were isolated from human peripheral blood using Lymphocyte Separation Medium (Solarbio). Monocytes were cultured in DMEM media supplemented with 10% FBS and GM-CSF (100 ng/ml, PeproTech) for 7 days to obtain M0 cells. IFN-γ (20 ng/ml, PeproTech) and LPS (20 ng/ml, Solarbio) were used to activate M0 cells. Morphology of PBMC derived macrophages was observed under a microscope and micrographs were taken.

### Tracing exosomes uptake by macrophages

Isolated exosomes were incubated with PHK26 Red Fluorescent dye (Sigma) for 10 min at room temperature. Labeled exosomes were washed with PBS and suspended in PRMI 1640 media. The labeled exosomes were added to THP-1 derived M0 cells and incubated for 3 h. Subsequently, cell conditioned media were removed, then fixed, permeabilized and stained with DAPI. Exosome uptake by macrophages was examined using a fluorescence inverted microscope.

### Western blot analysis

Cellular extracts or exosomes extracts were obtained using RIPA lysis buffer containing proteinase inhibitors and phosphatase inhibitors (Solarbio). After SDS-PAGE electrophoresis, electroblotting was conducted. The membranes were blocked and incubated with anti-TSP-1 antibodies (CST) or exosomes markers anti-CD63 antibodies (Proteintech), anti-CD9 antibodies (Proteintech). Specific antibody-bound protein bands were detected using ECL Plus reagent.

### CD4^+^T cells sorting and identification

Normal human PBMC were isolated from human peripheral blood using Lymphocyte Separation Medium and incubated for 2 h in 37°C/5% CO_2_. CD4^+^T cells were isolated using the human CD4^+^T cell isolation kit (BD Biosciences), following the manufacturer’s instructions. Cells were suspended in the supernatant with 10 μg/ml exosomes and added into 24-well plates precoated with anti-CD3 antibodies (10 μg/ml, Sino Biological). After 12 h, cells were collected. Th1 (CD4^+^ IFN-γ^+^), Th2 (CD4^+^ IL-4^+^), Th17(CD4^+^ IL-17^+^), Treg (CD25^+^ Foxp3^+^) cells were analyzed by flow cytometry.

### EdU incorporation

Cells were suspended in the supernatant with 10 μg/ml exosomes, incubated in culture media containing 20 mM EdU 12 h, and processed using the TransDetect® EdU Flow Cytometry Kit-647 Fluorophore (TransGen). Labeled cells were analyzed on a BD celesta.

### Flow cytometry analysis

Extracted exosomes 10 μg were incubated with 1 µl Aldehyde/Sulfate Latex Beads (Invitrogen) 15 min at RT. After washed, exosomes-beads complex were incubated overnight with anti-human TSP-1 antibody (Proteintech). Complex were washed with PBS and incubated with the secondary antibody (Biolegend) for 1 h at 4°C. The expression of TSP-1 of complex were detected by BD celesta flow cytometer.

After treatment with 10 μg/ml exosomes for 12 h, culture supernatant was collected. Inflammation cytokines secreted by THP-1 derived macrophages and PBMC derived macrophages and CD4^+^T cell were detected with LEGENDplex™ Human Inflammation Panel (Biolegend), following the manufacturer’s instructions. The Panel allowed for simultaneous quantification of 13 human inflammation cytokines: IL-1β, IFN-α2, IFN-γ, TNF-α, MCP-1 (CCL2), IL-6, IL-8 (CXCL8), IL-10, IL-12p70, IL-17A, IL-18, IL-23 and IL-33. Inflammation cytokines were measured on the BD celesta flow cytometer.

### Quantitative real-time PCR assay

Cultured cells were isolated using TRIzol reagent (Invitrogen) following the manufacturer’s protocol. cDNA was synthesized using the PrimeScript RT kit (TaKaRa). For real-time PCR analysis, mRNA expression was quantified using SYBR Green Premix Kit (TaKaRa).

### Statistical analysis

Multiple samples were compared by one-way analysis of variance to determine whether there were significant differences. The results were presented as the mean ± SD. *P* < 0.05 was considered statistically significant. All statistical data were analyzed using the GraphPad Prism 8 software.

## Results

### Expression of TSP-1 was up-regulated in tMSCs from *in vitro* LPS pretreatment

**Figure 1:**
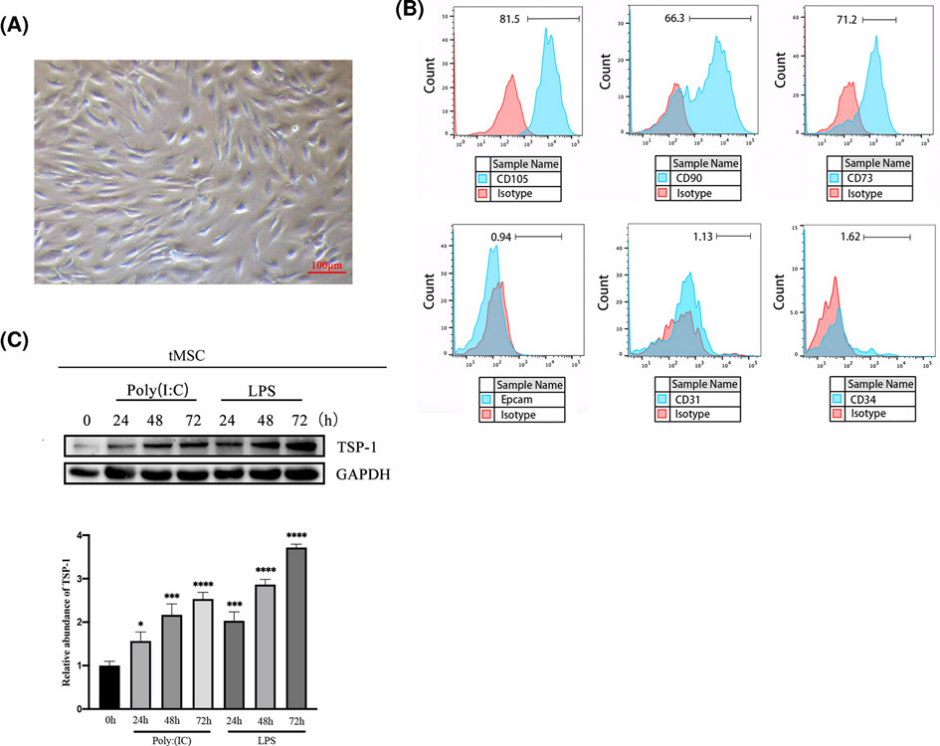
Expression of TSP-1 was up-regulated in tMSCs from *in vitro* inflammation treatment (**A**) Morphology of tMSCs isolated from normal thymus tissue, scale bar = 100 μm. (**B**) Flow cytometric detection of the cell surface markers of tMSCs and epithelial cell marker EpCam, endothelial cell marker CD31 and hematopoietic stem cell marker CD34. (**C**) tMSCs were stimulated by LPS or Poly (I:C) for 24, 48 and 72 h, using Western blot to detect TSP-1 protein expression and grayscale analysis. Data were represented as the mean ± SD of three independent experiments, *P<0.05, ***P<0.001, ****P<0.0001.

tMSCs were isolated from normal thymus and characterized. Cells exhibited typical fusiformis morphology (Figure 1A). Flow cytometry identification results demonstrated that it was positive for cell-surface markers of MSCs, CD105, CD90 and CD73, and thymus epithelial cell marker EpCam, endothelial cell marker CD31, and hematopoietic stem cell marker CD34 is negative (Figure 1B). MSCs pretreatment impacted their immunomodulatory functions. By detecting the expression of TSP-1, expression of TSP-1 significantly increased in the tMSCs pretreated with Poly (I:C) (50 μg/ml) or LPS (1 μg/ml) in a time-dependent manner. After 72 h treatment, the expression of TSP-1 is higher than 24 and 48 h treatment. (Figure 1C and Supplementary Figure S1). To determine the role of TSP-1 in thymic inflammation, we knocked down TSP-1 expression in pretreated tMSCs usingTSP-1 shRNA lentivirus (Supplementary Figure S2).

### MEXs^LPS^ can be used as a vehicle to express TSP-1

**Figure 2:**
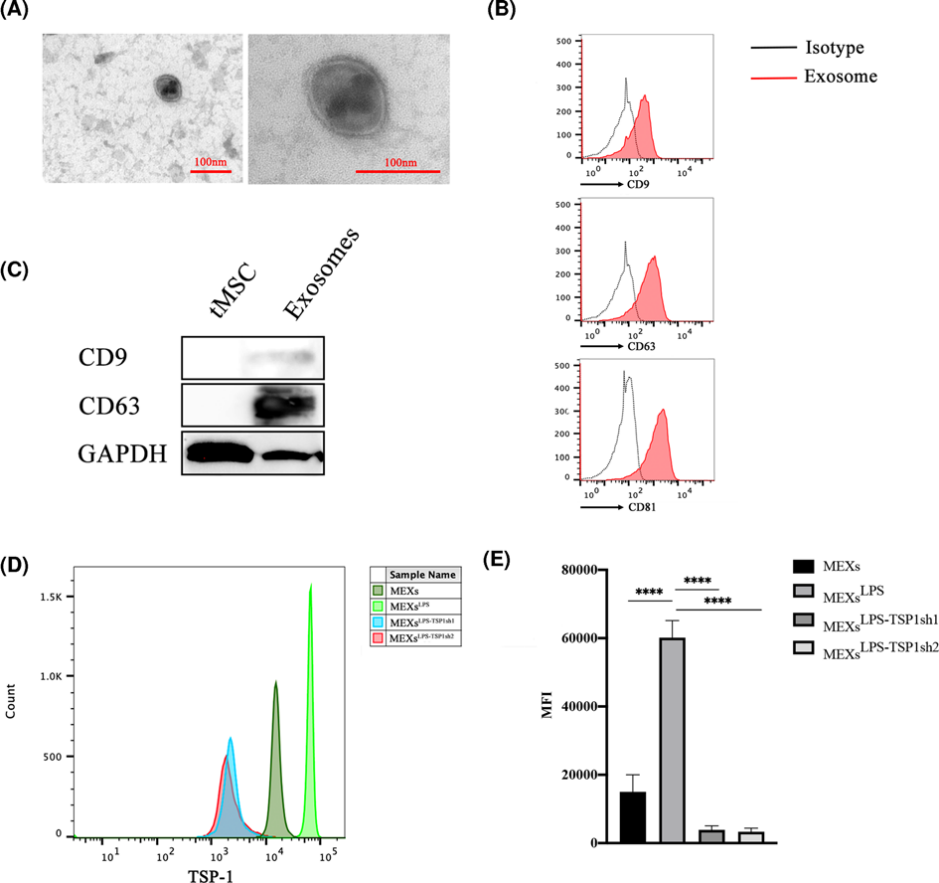
MEXs^LPS^ can be used as a vector to express TSP-1 (**A**) The particle size and morphology of MEXs detected by electron microscopy, scale bar = 100 nm. (**B**) Expression levels of CD9, CD63 and CD81 in MEXs detected by flow cytometry. (**C**) Expression levels of CD9 and CD63 in tMSCs and MEXs detected by Western blot. (**D** and** E**) Expression levels of TSP-1 in MEXs, MEXs^LPS^, MEXs^LPS-TSP1sh1^, MEXs^LPS-TSP1sh2^. Data were represented as the mean ± SD of three independent experiments, ****P<0.0001.

Here, we isolated tMSCs-exosomes (MEXs) from the supernatant of cultured tMSCs, then characterized their morphology and components. Electron microscopy revealed that MEXs were membranous vesicles ranging about 100nm in size and had a sphere-shaped morphology. (Figure 2A and see Supplementary Figure S2A for more vesicles version). Using flow cytometry, the identity of these particles was further confirmed to be exosomes, showing the presence of exosomal surface markers CD9, D63 and CD81 (Figure 2B). Western blot analysis also confirmed the expression of exosomes signature markers (Figure 2C). These findings affirmed that these nanoparticles were exosomes.

Cells can secrete different types of exosomes that have been classified according to their origin [41]. To determine whether TSP-1 was present in exosomes, we evaluated the expression of TSP-1 in MEXs^LPS^ and MEXs^LPS-TSP1sh^. Notably, higher and lower levels of TSP-1 were found in MEXs^LPS^ and MEXs^LPS-TSP1sh^ respectively (Figure 2D,E). This experiment implicated that the expression of TSP-1 in tMSCs or MEXs with comparable kinetics after same stimulation condition.

### MEXs-mediated TSP-1 promotes macrophage inflammation *in vitro*

**Figure 3:**
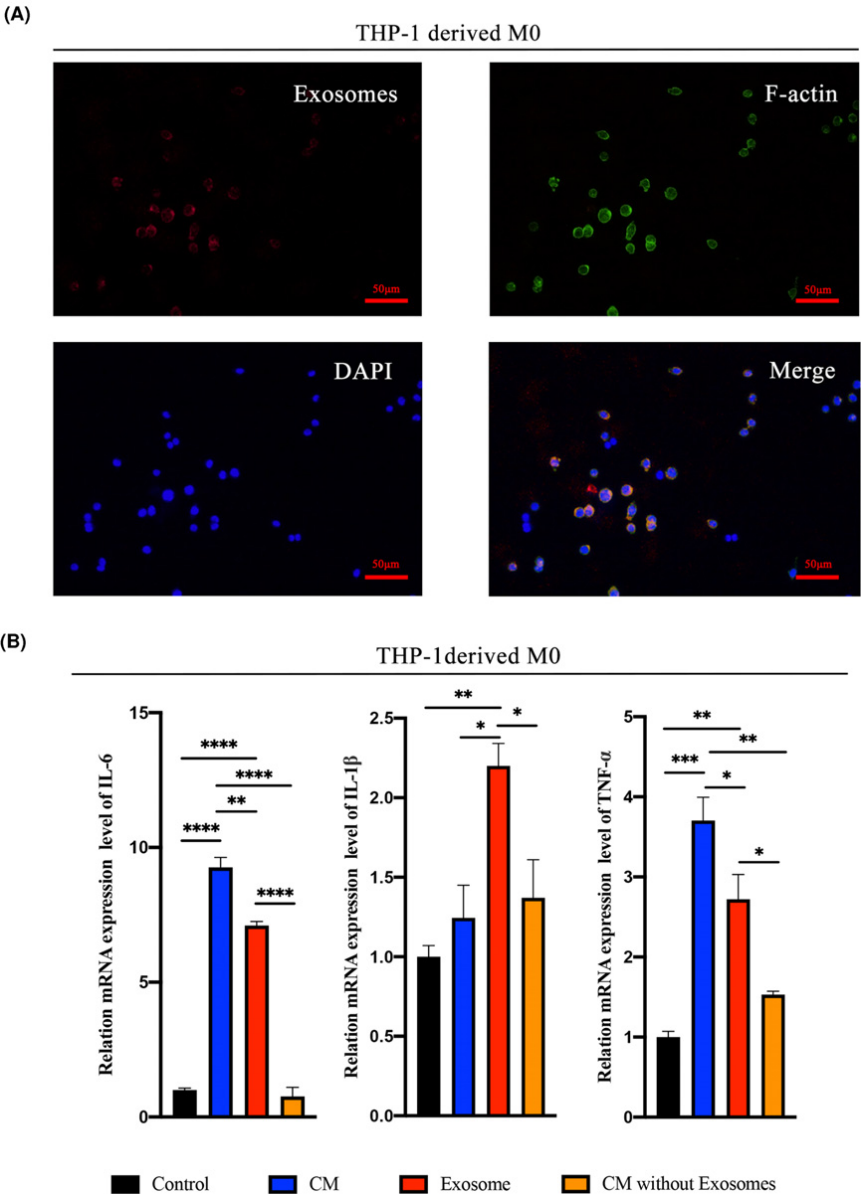
MEXs^LPS^ was the main inducer of inflammatory polarization in macrophages in the cell culture supernatant (**A**) Uptake of exosomes by THP-1-derived macrophages. Exosomes were labeled with Exo-PHK26 (red), macrophages were labeled with 488-Phalloidin (green) and nuclei of macrophages with DAPI (blue), bar = 50 μm. (**B**) Transcription levels of TNF-α, IL-1β and IL-6 in THP-1-derived macrophages educated by conditioned media (Control), LPS-tMSCs CM, exosome supernatant or conditioned media without exosomes from LPS-tMSCs CM for 24 h. Data were represented as the mean ± SD of three independent experiments, *P<0.05, **P<0.01, ***P<0.001, ****P<0.0001.

Monocytes and macrophages constitute an important cell group in inflammatory foci. They produce various pro-inflammatory cytokines, chemokines and play a key role in the immune response during infection [42]. A study revealed that macrophages could uptake exosomes rapidly [43]. Herein, we assessed the uptake of fluorescence-labeled exosomes using fluorescence microscopy. Notably, the uptake of LPS-tMSCs exosomes by THP-1-derived macrophages was evident (Figure 3A), suggesting that macrophages receive the biological signals from exosomes via direct uptake. Extracellular vesicles are also a major component in cell-conditioned media in addition to soluble factors (such as cytokines, chemokines and growth factors). Among these vesicles, exosomes have been regarded as the key mediators during intercellular communication [44]. To elucidate the functional effectors in exosomes, we used LPS-tMSCs conditioned media (CM), equal amount LPS-tMSCs exosome supernatant and CM without exosome to stimulate THP-1 induced macrophages. Comparatively, the mRNA expressions of M1 signature genes (IL-6 and TNF-α) were significantly highly up-regulated in the CM and MEXs^LPS^ group but not the exosome-free CM group. Although the expression of IL-1β in MEXs^LPS^ group was not significantly reduced in the CM, it was significantly up-regulated in MEXs^LPS^ group than exosome-free CM group. These findings highlighted MEXs^LPS^ as the main inducer of inflammatory polarization in macrophages in the cell culture supernatant (Figure 3B).

**Figure 4:**
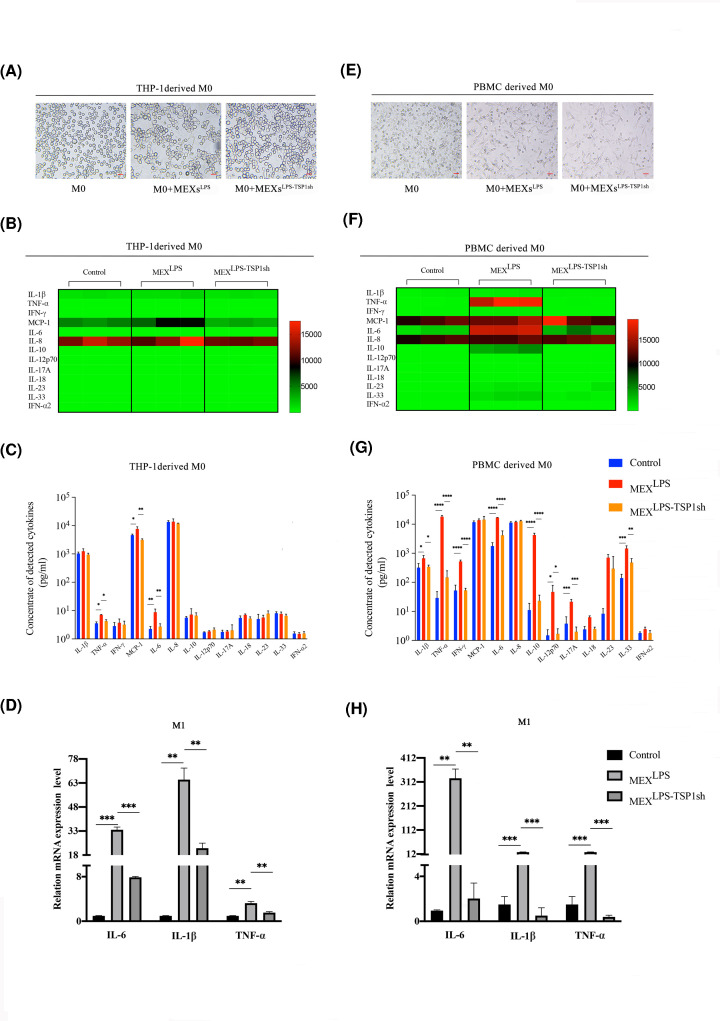
TSP-1 as the main factor in the proinflammatory regulation of macrophages (**A** and** E**) Morphological changes of THP-1-derived and PBMC-derived macrophages co-cultured with exosomes, bar = 20 μm. (**B** and **F**) Heat-map of cytokine expression profiles. THP-1-derived and PBMC-derived macrophages treated with MEXs^LPS^ and MEXs^LPS-TSP1sh^ for 12 h. All samples were run in triplicate. The colors illustrate fold changes (see color scale). Red: up-regulation; green: down-regulation. (**C** and **G**) Detection of inflammatory factors levels in culture supernatants of THP-1-derived and PBMC-derived macrophages treated with MEXs^LPS^ and MEXs^LPS-TSP1sh^ for 12 h. (**D** and **H**) Expression of IL-6, IL-1β and TNF-α in THP-1-derived and PBMC-derived macrophages when treated with MEXs^LPS^ and MEXs^LPS-TSP1sh^ for 12 h. Data are expressed as the mean ± SD from three experiments, *P<0.05, **P<0.01, ***P<0.001, ****P<0.0001.

In addition, the morphology and phenotype of THP-1-derived macrophages under MEXs^LPS^ and MEXs^LPS-TSP1sh^ treatments changed. On the other hand, the morphology of THP-1-derived M0 stimulated by MEXs gradually became fusiform and irregular (Figure 4A). The expression levels of M1-like macrophages related marker HLA-DR was significantly elevated after MEXs^LPS^ treatment, whereas HLA-DR and CD86 levels were lowered after MEXs^LPS-TSP1sh^ treatment (data not shown). Thereafter, we examined protein levels of 13 inflammation-related cytokines. Results showed that MEXs^LPS^ treatment significantly enhanced the secretion levels of IL-6, MCP-1and TNF-α. When we knocked down the expression of TSP-1, the secretion of these cytokines was significantly lowered (Figure 4B,C). Moreover, we examined transcriptional levels of M1 signature genes (IL-6, IL-1β and TNF-α) in THP-1-derived macrophages stimulated by MEXs^LPS^ and MEXs^LPS-TSP1sh^. The expression levels of M1 related genes exhibited a similar trend with the secretion of these cytokines (Figure 4D). These results indicated TSP-1 can promote the expression and secretion of inflammation-related cytokines by THP-1-derived macrophages via tMSCs exosomes.

Furthermore, when we analyzed the PBMC-derived macrophages, the morphology of PBMC-derived M0 stimulated by MEXs was gradually elongated and spindle-shaped (Figure 4E). MEXs^LPS^ treatment significantly enhanced the secretion levels of IL-6, IL-1β, IFN-γ, TNF-α, IL-10, IL-12p70, IL-17A, IL-23, IL-33. By knocking down the expression of TSP-1, the secretion of these cytokines was significantly reduced (Figure 4F,G). Also, expression levels of M1 related genes exhibited the same trend as secretion of these cytokines (Figure 4H).

In summary, MEXs^LPS^ promoted the expression and secretion of inflammatory cytokines including IL-6, IL-1β and TNF-α by macrophages, and TSP-1 plays a major regulatory role in this process.

### MEXs-mediated TSP-1 is a critical factor to differentiates CD4^+^T cells to Th1, Th17 *in vitro*

**Figure 5:**
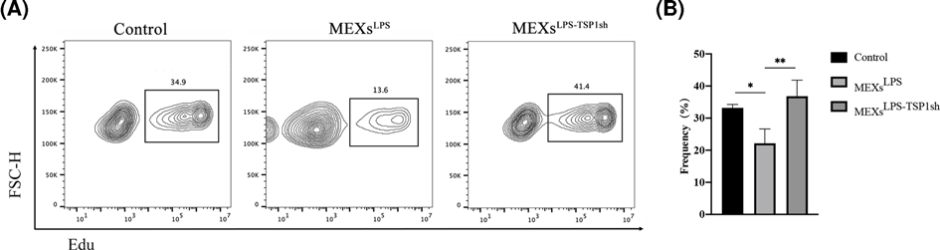
TSP-1 inhibition cell proliferation (**A**) Activated CD4^+^T cells were co-cultured with MEXs^LPS^ and MEXs^LPS-TSP1sh^ for 12 h, CD4^+^T cells were labeled with Edu-AF647 and detected by flow cytometry. (**B**) A representative image is shown. Data were expressed as the mean ± SD from three experiments, *P<0.05, **P<0.01.

Activated CD4^+^T cells cultured in supernatants with MEXs^LPS^ and MEXs^LPS-TSP1sh^ for 12 h, stained with Edu-AF647. We found that the proliferation of CD4^+^T cells was largely inhibited in the MEXs^LPS^ group. However, after MEXs^LPS-TSP1sh^ was added, the proliferation of CD4^+^T cells recovered, suggesting that TSP-1 inhibits CD4^+^T cell proliferation (Figure 5A,B).

**Figure 6:**
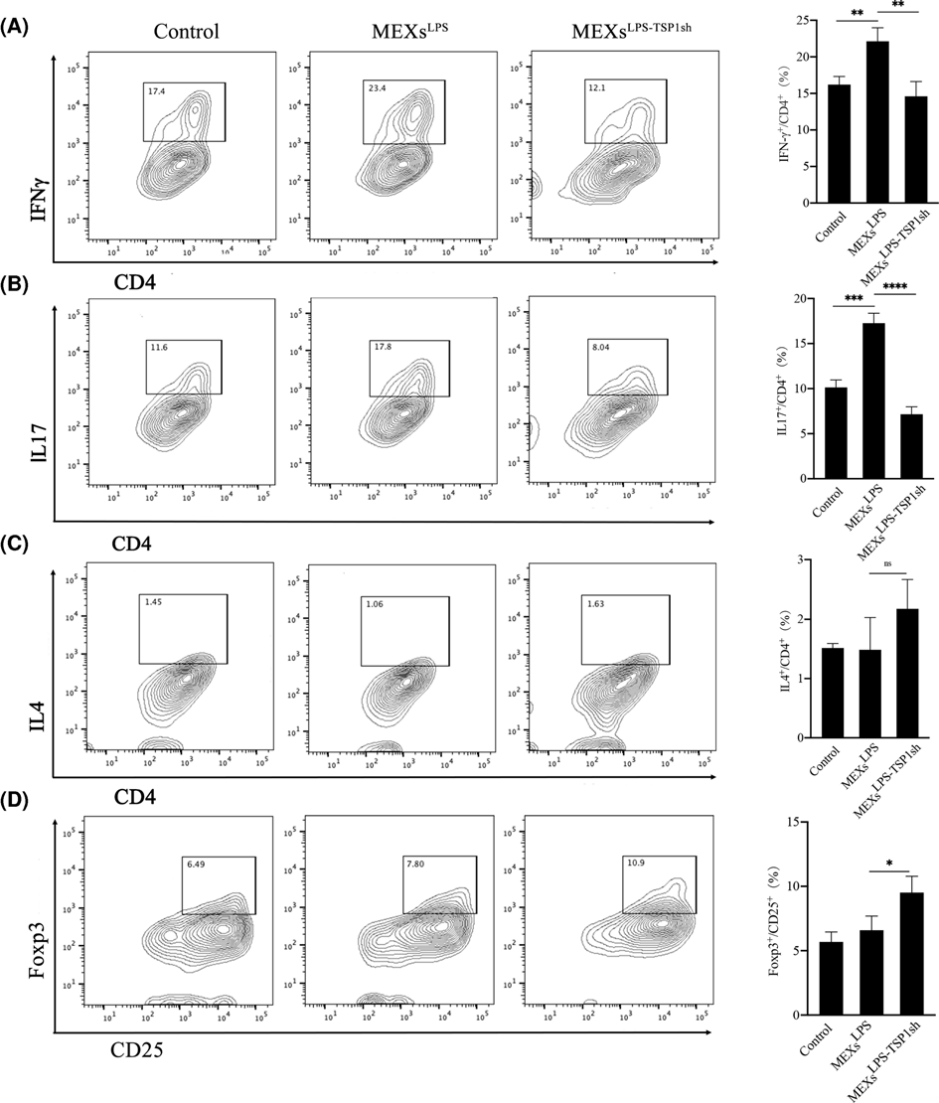
TSP-1 promotes differentiation of Th1 and Th17 cells and inhibits Tregs differentiation (**A**) Activated CD4^+^T cells were co-cultured with MEXs^LPS^ and MEXs^LPS-TSP1sh^ for 12hr, the percentage of Th1 (IFNγ^+^ CD4^+^T) cells was analyzed by flow cytometry. A representative image (left), statistical analysis of the data (right). (**B**) The percentage of Th17 (IL17^+^CD4^+^T) cells as analyzed by flow cytometry. (**C**) The percentage of Th2 (IL4^+^CD4^+^T) cells as analyzed by flow cytometry. (**D**) The percentage of Tregs (Foxp3^+^CD25^+^T) as analyzed by flow cytometry. Data are expressed as the mean ± SD from three experiments, *P<0.05, **P<0.01, ***P<0.001, ****P<0.0001.

Since TSP-1 significantly promoted inflammation, we sought to assess whether TSP-1 affects the differentiation of CD4^+^T cells. As expected, we found higher numbers of Th1, Th17 cells after the addition of MEXs^LPS^. Notably, MEXs^LPS-TSP1sh^ treatment inhibited Th1 and Th17 differentiation (Figure 6A,B), whereas it promoted Tregs differentiation (Figure 6D). However, it has no effects on Th2 differentiation (Figure 6C).

**Figure 7:**
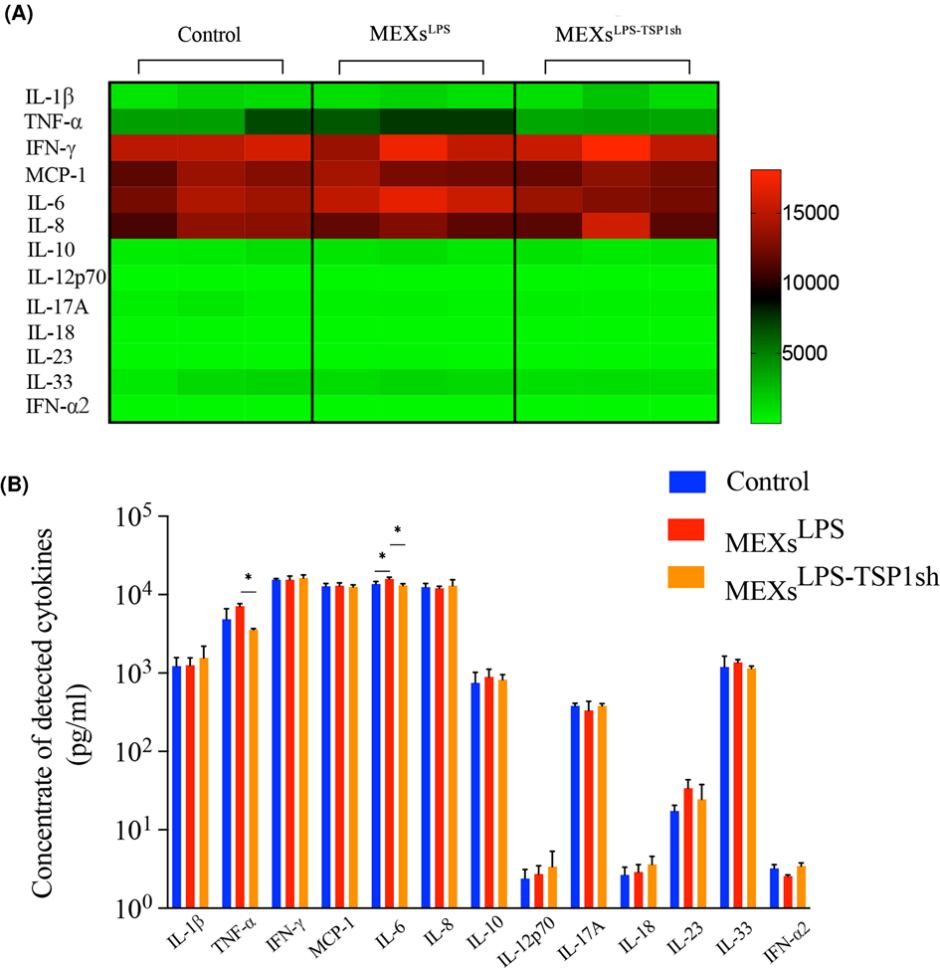
MEXs^LPS^ effectively promotes the secretion of inflammatory cytokines such as IL-6 and TNF-α by CD4+T cells (**A**) Heat map of cytokine expression profiles. CD4^+^T cells were treated with MEXs^LPS^ and MEXs^LPS-TSP1sh^ for 12 h. (**B**) Detection of inflammatory factors levels in culture supernatants of CD4^+^T cells treated with MEXs^LPS^ and MEXs^LPS-TSP1sh^ for 12 h. Data are expressed as the mean ± SD from three experiments, *P<0.05.

Furthermore, the inflammation-related cytokines in the cell culture supernatant were detected. MEXs^LPS^ significantly enhanced the secretion levels of IL-6. When we knocked down the expression of TSP-1, the secretion of IL-6 and TNF-α was significantly lowered (Figure 7A,B). Furthermore, TSP-1 could effectively promote the release of TNF-α and IL-6 in the co-culture system.

In summary, TSP-1 plays a critical role in regulating the inflammatory differentiation of T cells. MEXs^LPS^ inhibits T-cell proliferation, whereas it promotes Th1 and Th17 cell differentiation. On the other hand, the secretion of inflammatory cytokines, among them, IL-6 and TNF-α is elevated.

## Discussion

MSCs exert regulatory function through cellular contact and/or secretion of various regulatory factors [45]. However, the immune regulatory capacity of MSCs is induced by inflammatory factors such as cytokines in the inflammatory microenvironment [8,46]. Whether MSCs promote or inhibit inflammatory response relies on the inflammatory microenvironment. In most cases, MSCs show immunosuppressive regulatory function, and studies showed a lower number of T cells, impaired T-cell proliferation, and lower levels of proinflammatory cytokines IL-2, TNF-α, and IFN-γ in MSC-exosome treated mice [47]. Yet, in our study, LPS-tMSCs CM and exosomes exerted a proinflammatory regulatory effect, which promoted the expression of pro-inflammatory cytokines by macrophages and helper T cells. This may be attributed to the pretreatment method or the use of CM and exosomes instead of tMSCs. The above findings also reveal the plasticity of tMSCs immunomodulatory function.

In the past few years, researchers have suggested that TSP-1 has environment-specific pro-inflammatory and anti-inflammatory functions [16,48,49]. Exogenous TSP-1 enhances the TSP-1 secreted by HASMC stimulated by Poly(I: C) [50]. The enhanced gene expression of thrombospondin-1 (TSP-1) in human monocytic cells stimulated by Porphyromonas gingivalis LPS [51]. In our *in vitro* experiments, the expression of TSP-1 was elevated under the stimulation of LPS and Poly(I:C) in tMSCs in a time-dependent manner, affirming that TSP-1 participates in tMSCs response in an inflammatory stage. After knocking down the expression of TSP-1, TSP-1 expression in tMSCs and tMSCs exosomes was lowered, showing that tMSCs exosomes can function as a vehicle for expressing TSP-1. According to a study by Akyurekli et al. [52], TSP-1 derived from tumor cells exosomes triggered macrophages polarization to change the tumor inflammatory microenvironment primarily via the activation of p38, Akt and SAPK/JNK signaling pathways. In our study, the effect of LPS-tMSCs CM on macrophage polarization was significantly reduced when exosomes were eliminated, suggesting that exosome was the main form of CM that impacted on macrophage polarization. Moreover, when TSP1 in MEXs^LPS^ was silenced, macrophage secretion of IL-6, IL-1β and TNF-α was lowered. The inflammatory response in macrophages was rescued, further demonstrating that TSP-1 is the principal factor in the pro-inflammatory response of MEXs^LPS^ in macrophages.

The abundance of TSP-1 has been reported to be inversely related to the proliferation of certain cell types [53]. Soluble TSP1 was a dose-dependent inhibitor of Jurkat T-cell proliferation [54]. However, the mechanism by which TSP-1 inhibits proliferation remains elusive. In this experiment, MEXs^LPS-TSP1sh^ highly promoted the proliferation of CD4^+^T cells compared to MEXs^LPS^ and showed the inhibitory effect of TSP-1 on CD4^+^T cell proliferation.

Th1 and Th17 cells are associated with several autoimmune diseases as they induce pro-inflammatory mediators and recruit immune cells to the inflammation sites [55]. It is also proposed that TSP that is produced in the lymph node may convert naive CD4^+^CD25^−^ T cells into Tregs that negatively control the inflammatory processes either in lymphoid organs and/or in sites of inflammation [56]. We demonstrated that MEXs^LPS^ effectively promoted the differentiation of CD4^+^T cells to Th1 and Th17 and inhibited Tregs differentiation, and the secretion of IL-6 was significantly enhanced. However, after TSP-1 was silenced, the secretion of IL-6 and TNF-α was lowered. However we can ignore the possibility that homologous stromal cells added to the co-culture system produced IL-6, thus the exosomal transfer of TSP-1 could still be regarded as the primary factor that regulates the differentiation of Th1 and Th17 cells. Of note, IL-17A and IFN-γ levels were not significantly increased in the cultured supernatant, although Th1 and Th17 differentiation were evident in the MEXs^LPS^ group. We speculated that this could be related to the time point of stimulation.

Moreover, MSCs regulate the immune system in two aspects, for instance, studies have shown that they can promote the function of Treg cells, thus can either directly or indirectly play a role in immunosuppressive regulation [57,58]. The current understanding of the immunomodulatory function of human tMSCs is limited. Since tMSCs exist in the environment of development of T cells and Treg cells, additional in-depth studies are required to assess whether it is different from umbilical cord blood-derived MSCs and bone marrow-derived MSCs in terms of immunomodulation. In this study, the generation of Treg cells was promoted via TSP-1 was silencing, this also illustrates the pro-inflammatory effect of TSP-1.

In conclusion, findings from this work demonstrated that TSP-1, as the main factor of exosomes derived from LPS-treated tMSCs, contributes to the polarization of M1-like macrophages and differentiation of CD4^+^T cells to Th1 and Th17. This indicates that exosomal transfer of over-expression of TSP-1 from tMSCs could promote immune cell inflammation.

## Supplementary Material

Supplementary Figures S1-S2

## Data Availability

The data used to support the findings of this study are available from the corresponding author upon request.

## Abbreviations

DAPI: 4′6-diamidino-2-phenylindole
EdU: 5-ethynyl-2′-deoxyuridine
GAPDH: glyceraldehyde-3-phosphate dehydrogenase
IL: interleukin
PBMC: peripheral blood mononuclear cell
RT-qPCR: real-time quantitative PCR
shRNA: short hairpin RNA
TNFα: tumor necrosis factor α
