# Supplementary material for: Exosomes derived from LPS-stimulated human thymic mesenchymal stromal cells enhance inflammation via thrombospondin-1
Source: Biosci Rep. 2021 Oct 12;41(10):BSR20203573. doi: 10.1042/BSR20203573 (PMC8521535; doi:10.1042/BSR20203573)
Supplement: Supplementary Figures S1-S2 [file BSR-2020-3573_supp.pdf]

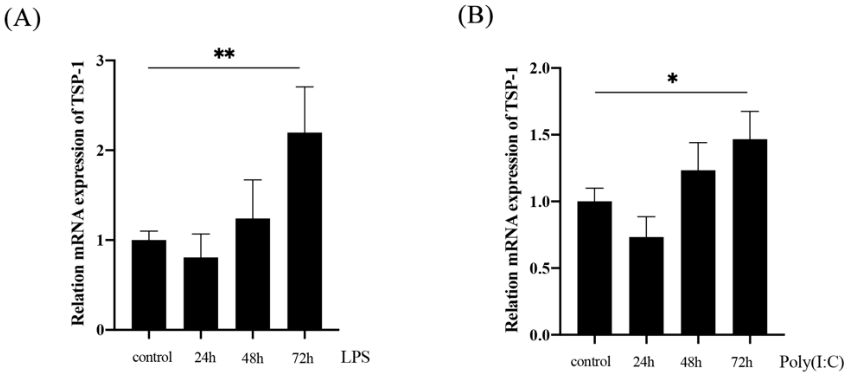

Supplementary 1. LPS and Poly(I:C) up-regulated TSP-1 expressed by tMSC.(A)Pretreated tMSCs were treated with LPS for24, 48 and 72hr, using qRT-PCR to detect TSP-1 expression.(B)Pretreated tMSCs were treated with Poly(I:C) for24, 48 and 72hr, using qRT-PCR to detect TSP-1 expression. Data were represented as the mean ± SD of three independent experiments.*p< 0.05, **p< 0.01, ***p< 0.001.

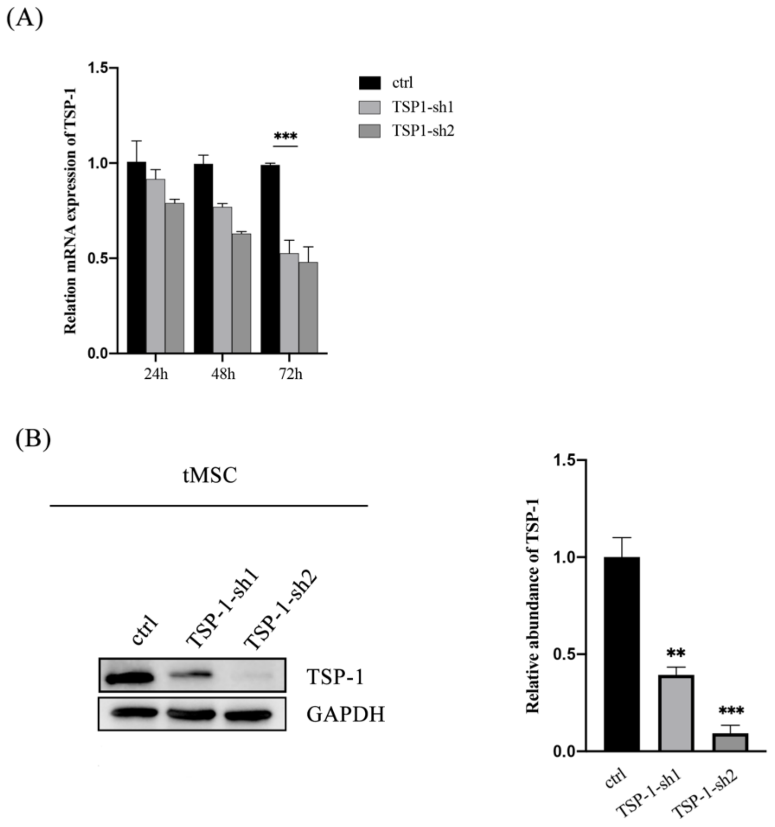

Supplementary 2. TSP-1 lentivirus down-regulated TSP-1 expressed by tMSC.(A)Pretreated tMSCs were treated with TSP-1-shRNA lentivirus for24, 48 and 72hr, using qRT-PCR to detect TSP-1 expression.(B)Pretreated tMSCs were treated with TSP-1-shRNA lentivirus for 72hr, using western blot to detect TSP-1 protein expression (left) and grayscale analysis (right).Data were represented as the mean ± SD of three independent experiments.*$p < 0.05$, **$p < 0.01$, ***$p < 0.001$.

Electron microscopy of Exosomes (more vesicles versions)

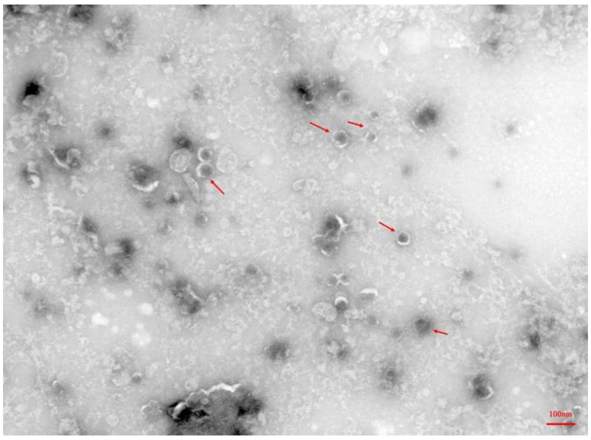

Supplementary Figure2A Electron microscopy of Exosomes. Electron microscopy image of isolated exosome (red arrow) derived from tMSC.Scale bar = 100 nm.
